# “Sometimes I’m interested in seeing a fuller story to tell with numbers” Implementing a forecasting dashboard for harm reduction and overdose prevention: a qualitative assessment

**DOI:** 10.1186/s12889-025-22004-y

**Published:** 2025-03-07

**Authors:** Jesse Yedinak Gray, Maxwell Krieger, Alexandra Skinner, Samantha Parker, Melissa Basta, Nya Reichley, Cathy Schultz, Claire Pratty, Ellen Duong, Bennett Allen, Magdalena Cerdá, Alexandria Macmadu, Brandon D.L. Marshall

**Affiliations:** 1 Department of Epidemiology, Brown University School of Public Health, 121 South Main Street, Box G-S-121-2 Providence, Providence, RI 02912 USA; 2 State of Rhode Island Department of Health, Providence, RI USA; 3 State of Rhode Island Executive Office of Health & Human Services, Cranston, RI USA; 4 Center for Computation & Visualization (CCV), Brown University, Providence, RI USA; 5 Center for Opioid Epidemiology and Policy, NYU Grossman School of Medicine, New York, NY USA

**Keywords:** Overdose, Harm reduction, Surveillance, Data dashboard, Forecasting, Predictive analytics, Machine learning, EPIS, Data modernization

## Abstract

**Objectives:**

The escalating overdose crisis in the United States points to the urgent need for new and novel data tools. Overdose data tools are growing in popularity but still face timely delays in surveillance data availability, lack of completeness, and wide variability in quality by region. As such, we need innovative tools to identify and prioritize emerging and high-need areas. Forecasting offers one such solution. Machine learning methods leverage numerous datasets that could be used to predict future vulnerability to overdose at the regional, town, and even neighborhood levels. This study aimed to understand the multi-level factors affecting the early stages of implementation for an overdose forecasting dashboard. This dashboard was developed with and for statewide harm reduction providers to increase data-driven response and resource distribution at the neighborhood level.

**Methods:**

As part of PROVIDENT (Preventing OVerdose using Information and Data from the EnvironmeNT), a randomized, statewide community trial, we conducted an implementation study where we facilitated three focus groups with harm reduction organizations enrolled in the larger trial. Focus group participants held titles such as peer outreach workers, case managers, and program coordinators/managers. We employed the Exploration, Preparation, Implementation, Sustainment (EPIS) Framework to guide our analysis. This framework offers a multi-level, four-phase analysis unique to implementation within a human services environment to assess the exploration and preparation phases that influenced the early launch of the intervention.

**Results:**

Multiple themes centering on organizational culture and resources emerged, including limited staff capacity for new interventions and repeated exposure to stress and trauma, which could limit intervention uptake. Community-level themes included the burden of data collection for program funding and statewide efforts to build stronger networks for data collection and dashboarding and data-driven resource allocation.

**Discussion:**

Using an implementation framework within the larger study allowed us to identify multi-level and contextual factors affecting the early implementation of a forecasting dashboard within the PROVIDENT community trial. Additional investments to build organizational and community capacity may be required to create the optimal implementation setting and integration of forecasting tools.

**Supplementary Information:**

The online version contains supplementary material available at 10.1186/s12889-025-22004-y.

## Background

Significant investments from the United States (US) Centers for Disease Control and Prevention (CDC) in data modernization and systematic data collection have vastly improved overdose surveillance data quality and availability across the country [1, 2]. In the US, efforts to curb the escalating overdose crisis rely on data products such as hotspot maps, data dashboards, and improved case definitions from local, state, and national overdose surveillance datasets (e.g., overdose deaths, emergency department visits, and ambulance runs) [1]. Community-level data surveillance tools and dashboards are increasing across the country, offering early insights into the approaches required to adapt such tools to community-driven systems of overdose prevention and harm reduction service delivery [3, 4]. Despite widespread efforts to improve the timeliness, interpretation, completeness, and accessibility of overdose data, the use of these tools varies by region [1, 5, 6]. Furthermore, the institutions generating these overdose data products have yet to fully capture the persistent racial and ethnic disparities that exist across our systems of care, including our death identification and classification systems [7]. Finally, rapidly changing dynamics of the unregulated drug supply have precipitated the latest surge in overdose deaths within the US, making time delays and lags in surveillance data a significant barrier to evidence-informed decision-making about resource allocation [8–11]. These concerns point to a need for timely, multi-dimensional data sources that capture the geographical makeup, inequitable access to resources, and longstanding policy decisions that contribute to the complexity of our regional responses to the overdose crisis.

Nationally, top policy and research experts are demanding solutions to the issues plaguing the data-driven response to the overdose crisis and are pushing for innovations that include machine learning methods and dashboarding [12]. For example, the CDC has been evaluating the application and successful use of forecasting for influenza detection and preparedness since 2014 [13]. Forecasting using machine learning or predictive analytics also offers potential as an innovative tool to proactively guide responses to the overdose crisis in the US [14]. First, such methods could enhance existing overdose surveillance efforts by leveraging additional datasets that better capture structural vulnerabilities in under-resourced neighborhoods. Second, by applying a wider set of predictors, researchers can use these novel methods to forecast both persistent and emerging hotspots in smaller regions (i.e., neighborhoods) at future risk for increased overdose activity [14]. Third, researchers have highlighted the need to address the overdose crisis by responding to emerging characteristics of regional clusters of overdose and local patterns of drug use [15]. Forecasting methods offer local organizations and health departments the tools necessary for a data-driven response at the neighborhood level. Finally, such methods may support overdose prevention and harm reduction efforts *prior to* hotspots developing [16].

Previously, our research team has worked closely with community and state-agency stakeholders and harm reduction partners in Rhode Island to increase the acceptability and use of a curated, online data dashboard for overdose surveillance starting in 2015 [17–19]. We then piloted the feasibility of mapping-driven predictive analytics to address future risk for regional outbreaks of overdose and infectious diseases, where we collaborated closely with key community stakeholders [20]. Continuing that work, we built and validated a machine learning forecasting tool and interactive dashboard to identify areas at risk for increased overdose activity and potentially direct local harm reduction resources to areas of prioritized need. The latter study, named PROVIDENT (Preventing OVerdose using Information and Data from the EnvironmeNT), was launched as a randomized community trial within the state of Rhode Island in 2021 [21]. Through our prior work creating and implementing data dashboards and predictive mapping tools, we recognized that the PROVIDENT framework is a new model for data-driven harm reduction service delivery that deserves further study. Specifically, the PROVIDENT project aims to support the ongoing and strategic use of predictive data in harm reduction organizations, including planning for future overdose risk and data-driven resource allocation by neighborhood.

Within the larger parent trial, we piloted an implementation study to understand individual, organizational, and community capacity within community-based harm reduction organizations for interacting with the PROVIDENT forecasting intervention and dashboard [21]. Given that the goal of the PROVIDENT forecasting dashboard was focused on leveraging partnerships and action across a network of statewide, nonprofit harm reduction service agencies and other key stakeholders, we aimed to assess the multi-level influential factors at play during the early phases of intervention implementation. The Exploration, Preparation, Implementation, Sustainment (EPIS) Framework offers a multi-level analysis (i.e., inner and outer environmental factors) that is unique to public sector implementation and makes space for assessing barriers and facilitators of uptake across four phases of implementation [22]. This study focused on the first two EPIS phases, exploration and preparation, to better understand the inner contextual factors (e.g. within-organization use of data, resource needs, readiness to change) and outer environment (e.g. funding environment, community network) that influenced the early development and uptake of the PROVIDENT forecasting dashboard among harm reduction service providers.

## Methods

### Design and objectives

This research study was designed as an implementation study within a larger statewide randomized community trial, PROVIDENT (NCT05096429) [21], approved by the Brown University IRB 1,910,002,566. The trial protocol and other details have been published previously in detail [21]. In brief, in collaboration with the state health department, we developed a forecasting model to identify neighborhoods (operationalized as census block groups) at the highest risk of future fatal overdose. The model has been previously validated and consistently predicts the top 20th percentile of neighborhoods within which at least 40% of overdoses occur over a subsequent six-month period [23]. Next, predictions were visualized in an accessible online forecasting dashboard, and we contracted with multiple harm reduction organizations to incorporate the forecasts into their decision-making about resource allocation (e.g., where to conduct naloxone distribution and street outreach). The PROVIDENT forecasting dashboard contains detailed neighborhood-level information, including: (1) predictive results indicating whether a neighborhood has been identified by the PROVIDENT model as being at high risk of fatal overdoses over the next six months; (2) social vulnerability indicators at the neighborhood, city, and state levels describing housing and economic resources (e.g. percentage of residents who live alone, percentage of residents with rent burden greater than 30% of income); and (3) locations of interest for a particular neighborhood, which utilizes foot traffic data as a proxy for areas where people tend to congregate. Additionally, the PROVIDENT forecasting dashboard provides interactive features such as two data collection forms for harm reduction organizations: (1) a *Neighborhood Rapid Assessment* to capture existing resources and potential barriers to service, and (2) a *Six Month Resource Plan* to track future resource planning and relationship-building efforts at the neighborhood level.

We recruited organizations to participate in the larger PROVIDENT trial by contacting all statewide harm reduction organizations responsible for distributing the state’s harm reduction supplies. The three participating organizations were compensated with a contract for $200/hour (up to 50 hours or $10,000 USD annually) for their teams’ attendance at our ‘Data Academy’ workshops and follow-up sessions. Specifically, to train peer outreach workers, case managers, and program coordinators/managers on the forecasting model and PROVIDENT forecasting dashboard, we created a series of interactive and highly accessible workshops nicknamed “The Data Academy.” The goal was to establish a foundation of public health data literacy skills and common language among dashboard users to improve confidence in data-driven decision-making. These workshops started as ten hours of training on topics such as counts, rates, and percentages, how and when to use overdose data, and how to read data charts and maps. Our team has previously published our approach as an instructional guide for other regions looking to improve the accessibility of their dashboards for wider audiences, where we offer self-assessment questions for vetting, displaying, and contextualizing overdose data [24]. The research team also hosted ongoing dashboard improvement meetings for real-time feedback and improvements to the dashboard. Organizations that attended the Data Academy workshops also received iPads for their participation to facilitate the use of the PROVIDENT forecasting dashboard in their outreach and field-based work. All individuals granted access to the PROVIDENT forecasting dashboard were staff from community-based organizations, agreed to training on the tool, and signed a confidentiality agreement not to share the information with law enforcement. Visualized data at the neighborhood level were guarded with caution and care so as not to be used to perpetuate or increase policing, profiling, or other punitive surveillance responses [25]. Harm reduction organizations were asked to log into the PROVIDENT forecasting dashboard at least once every six months to view the neighborhoods forecasted at higher risk, and to complete at least one Neighborhood Rapid Assessment form every six months for a neighborhood of their choice.

Because of the attention paid to enrolling and onboarding participants at the organizational level in the PROVIDENT trial, focus groups were chosen as an efficient approach to understanding within-organization responses to participation in the intervention at the six-month baseline. We conducted a series of focus groups with staff at harm reduction organizations who had previously agreed to participate and enroll in the PROVIDENT trial. Examples of discussion prompts included asking about organizational capacity and funding, data and reporting burden from funders (e.g. naloxone kits or harm reduction supplies distributed, referrals to care, case management services, etc.), the use of surveillance data tools such as hotspot maps, and the overall culture and resources for staff and programming at each organization. We also sought to understand whether our team’s supplemental Data Academy workshops and related partnership efforts contributed to increasing the organizational or individual uptake of the PROVIDENT intervention.

### Organizational participants

We contacted all statewide harm reduction organizations responsible for distributing harm reduction supplies, including sterile syringes/needles, fentanyl test strips, and naloxone, through fixed-site and mobile/outreach distribution. The peer outreach workers at these harm reduction organizations are also responsible for collecting individual-level data for all harm reduction services and interactions provided in Rhode Island (e.g., clients are asked questions such as personal patterns of drug use and injecting behaviors, housing status or location, need for additional services). Initially, four harm reduction organizations were recruited and were under contract as partners in the PROVIDENT trial, and we had fifteen staff members as primary contacts from those four organizations. However, one organization left the study prior to focus group enrollment due to a shift in programming/staffing to different high-demand services. This left ten staff members from three statewide harm reduction organizations to serve as primary contacts for the implementation study. Each of the remaining non-profit community-based organizations in our study provide services locally in Rhode Island for people who use drugs. Our three partnering organizations are peer-led by roughly 15–45 staff members and operate fixed and mobile outreach programs across the state.

### Baseline focus groups

Focus groups (*n* = 3) were held in person at the three organizations (with each focus group corresponding to a distinct organization) approximately six months after the initial dashboard launch. Focus groups were held between April 2022 and June 2022. Participants were eligible for a focus group if their employer offered peer support and harm reduction services to prevent overdoses and if their employer had previously agreed to engage with the PROVIDENT study and forecasting dashboard starting in November 2021. The focus group participants (*n* = 15) held staff roles in their respective organizations, including peer outreach workers, case managers, and program coordinators/managers; demographic data was not collected from participants to increase confidentiality in a small sample. Participants in the focus groups did not need to have participated in any prior Data Academy workshops, as staff members within each organization were allowed to train their coworkers on how to access and utilize the forecasting dashboard. Focus group recruitment occurred through email, sent to the ten primary staff members at the three organizations. From there, those contacts could invite additional participants through word-of-mouth or forwarding the email to interested colleagues. Of the original ten primary contact staff initially invited to participate, four chose not to participate for unknown reasons. Participation in the focus groups was voluntary. Participants were compensated $50 cash for up to ninety minutes, which included time for informed consent procedures. All participants provided informed consent. Focus groups ranged in size from 3 to 8 participants, and the average duration was approximately one hour. Focus groups were facilitated by two management-level staff members of the PROVIDENT research team with more than five years of prior survey and qualitative research experience in harm reduction (JYG, first author, cisgender female, master’s degree; MK, second author, cisgender male, bachelors degree), and prior experience working in a community harm reduction setting (JYG). Additional staff members were present for logistical support (CP, eighth author, cisgender female, master’s degree; JEG, nonbinary, master’s degree). There were no other non-participants present for the focus groups. The focus group facilitators had previously met the participants during the Data Academy or through prior research studies in Rhode Island, and participants were aware of the goals of the PROVIDENT dashboard and the research team’s prior work in Rhode Island. The focus group was facilitated with a guide and prompts developed for this study to elicit conversation across three categories relevant to the implementation environment: (1) organizational culture around data, (2) testing the web tool, and (3) team culture and resources [see Additional file 1]. The focus group guide was not tested with participants prior to use. No field notes were taken or included in the analysis, and repeat focus groups were not conducted. Focus groups were audio recorded, and audio recordings were transcribed by the company Daily Transcription; transcripts were coded and managed in Dedoose qualitative coding software [26].

Two members of the research team who had prior experience with qualitative analysis served as primary coders and analyzed transcripts using inductive and deductive approaches (AS, SP) [27]. The initial codebook was generated inductively: two coders conducted parallel coding and were blinded to participant and focus group identity. Next, they resolved discrepancies from the blinded coding through repeated active reading of transcripts for codes that arose most frequently and strongly [28]. The transcripts were then deductively coded through the lens of the EPIS framework [22]. Codes were iteratively revised, expanded, and merged as needed. Codes were first applied at the individual level to the transcripts and then reviewed at the focus-group level for across-group application of the codes and divergent cases. Discrepancies in coding were resolved by consensus in recurring meetings of the coders, and the codebook was periodically refined to accommodate emergent themes [27]. A third member of the research team (AM), a faculty member with experience in qualitative analysis and research in harm reduction, reviewed the results and provided support in finalizing the codebook. The final codebook included all themes and codes, with descriptions of how the codes were operationalized [see Additional file 2].

Current literature on qualitative data analysis suggests a minimum of four focus groups to meet saturation and reliably identify all potential themes [29]. However, the literature also indicates saturation may be met with fewer than four focus groups, particularly when the focus group guide and recruitment criteria are highly focused, as in the case of this study [30, 31]. Therefore, we feel data saturation was met for the two primary themes, given the depth of examples across the three focus groups. Primary codes within each of the first two themes were applied most frequently and consistently across the three focus groups, with a range of 12–40 applications. Considering the nature of the EPIS exploration and preparation phases, we did not apply a quantitative threshold for saturation. Some codes within the third theme were less frequent in comparison (fewer than 10 applications each), indicating the opportunity for further exploration of this theme in future work.

## Results

Three primary themes emerged from the focus groups, based on frequency and depth of discussion as assessed by primary codes and subcodes, and elucidated multiple factors influencing the implementation environment of the PROVIDENT forecasting dashboard. These themes broadly mapped onto the EPIS framework, including: (1) the inner context characteristics affecting organizations, such as concerns about capacity and burnout; (2) the outer factors of community influence, including client advocacy and data collection demands; and finally, (3) characteristics of the intervention and how PROVIDENT fits with the intended audience.

### Inner context elements: concerns about capacity, burnout, and trauma

The EPIS framework highlights within-organization elements that create the “inner context” of the implementation environment, such as the use of data, resource needs, attitudes toward a new intervention, and readiness to change [32]. Within each focus group, participants described the broad range of harm reduction services delivered by their organizations across Rhode Island. Participants then offered insights into their ability to maintain and/or expand these services, and whether or not they were using emerging data and surveillance tools (including PROVIDENT) to inform their work and decision-making. Two sub-themes emerged within this context, including organizational considerations around staffing for new data or intervention efforts and the emotional labor of direct service delivery within these environments.

The theme of limited staffing and covering multiple roles was echoed across the three focus groups, along with the need for increased technology, space, and resources to support the organizations’ work. Participants reported that these organizational constraints interfered with their abilities to collect data efficiently and to engage thoughtfully with the dashboard.

“*It would be nice to have a broader support system. Meaning*,* like…provide us materials that would increase the way we do our data. Like*,* more computers*,* more people*,* and things like that*,” *FG 1*,* Participant 3.*

Participants noted that the lack of resources and staff limited the ability of outreach workers to do the data collection related to their roles, saying a factor “*that causes that burnout fatigue is like*,* you know*,* give us resources…You’ll get more accurate [data] and we’re going to stop playing the cat-and-mouse game*,” *FG 1*,* Participant 4.*

Participants also reported performing several roles within the organization: “*So*,* you can come in with maybe two hats*,* but at the end of the year*,* you know*,* you’re wearing four*,” *FG 3*,* Participant 2.*

Chronic stress that inhibits staff from performing even their basic job duties presents a barrier to adopting additional data-related tools or responsibilities. One participant spoke to the challenges of face-to-face outreach and the toll it takes on the organization and its staff without proper support: “*I get traumatized working with these clients. When they fall off*,* or they die*,* that’s a problem for me*,* you know*,” *FG 3*,* Participant 2.*

Some participants emphasized taking time off to manage mental health if work becomes overwhelming:

“*What’s important is*,* in order for us to do our job the way they want us to do it*,* and the way we were hired to do it*,* we have to be okay here first*,* [in our heads]*,” *FG 3*,* Participant 3.*

Participants also discussed organizational responses to burnout concerns among staff:

“*Our [organization]…is real big on making sure that us workers have all the trainings*,* and all the resources that we need to keep going through what we go through. Trying to help the people that we help…And we have more capability now to reach more people*,* and handle their stresses*,* and manage our own*,” *FG 3*,* Participant 2.*

Staff face competing priorities as they balance their own stress and burnout with their clients’ most direct needs, which may leave little opportunity to engage with broader community-level forecasting tools or outreach. Participants described navigating structural barriers and struggling to get resources for their clients, as contributing to burnout among staff.

“*You have a client*,* and you try so many different avenues to help them and no matter-you’re always hit with a brick wall. Like*,* you’re always–you can never accomplish what you want–like to even get to the n-never mind the next step. You can’t even finish your first step*,” *FG 1.*

### Outer-Context demands: the invisible labor of advocacy and data collection demands

The EPIS framework notes that the “outer context” of the implementation environment includes socio-political factors, advocacy, and the overall funding environment [22]. Each focus group described their data collection responsibilities as part of their overall funding requirements and discussed the sensitive nature of collecting personal data from their clients in exchange for services. All three groups stressed the importance of building trusting relationships with their clients to facilitate better data collection when delivering harm reduction services, particularly when asking for identifiable personal information such as current drug use, injecting history, or housing status.

Discussion focused on their approach with clients as follows:

“*‘Cause we do ask a lot of things…sometimes if you have a consistent client*,* you already kind of know their background and their needs. And sometimes*,* I know some of the questions that we have [to ask]. Like*,* hey*,* do you engage in sex work? And that can be a little bit intimidating to ask. Especially if you’re doing outreach and there’s a bunch of people around*,* because you don’t want everybody to know this person is doing sex work*,*” FG 3*,* Participant 4*.

Participants similarly described how building rapport with clients requires time and trust but eventually facilitates data collection:

“*The subject matter that we’re talking about is a sensitive topic. So you need to invest that time in people so they’re comfortable enough with you so you can get that inside scoop and that’s kind of*,* like*,* where I am…You know*,* especially ‘cause just the overall stigma of a person who injects drugs*,* you know. They’re always wary like*,* what can I say*,* what should I not say?… All that time you’re investing that you’re not necessarily getting anything right away but your goal is to hopefully get that valuable data in the future*,” *FG 1.*

All focus groups addressed the emotional or unseen labor of relationship building, particularly because delivering harm reduction services involves asking highly personal questions about drug use patterns and other behaviors:

“*Sometimes I’m interested in seeing a fuller story to tell with numbers ‘cause it does suck to*,* like*,* at the end of the month give a numbered report*,* when I’m*,* like*,* oh*,* but so much happened that’s so not captured in this because it’s just quantitative… I feel like we can capture more ‘cause I would like to see more*,* and I know we could tell a bigger story with it*,* but I think we’re in a sweet spot right now*,” *FG 2*,* Participant 4.*

*“In the beginning*,* when you go to ask them about a couple of questions*,* you know*,* they tend to be*,* uh*,* defensive at first*,* you know…That’s where your technique comes in*,* you know. You give ‘em a little joke or something*,* you ask ‘em a couple questions*,* and it makes it a-a lot easier…I mean*,* you gotta do the data collection*,* and you just gotta find a way to make it*,* uh*,* not enjoyable*,* but bearable*,* I guess. So*,* that’s what we’re trying to do*,*” FG 3*,* Participant 3.*

### Characteristics of the intervention: relationships and usability

The concept of “fit” of the intervention within EPIS helps describe intervention features and their appropriateness for the given setting and acknowledges the need to adapt the intervention for optimal fit [32]. Additional community-level factors in Rhode Island include the organizational relationships with other organizations and the Rhode Island Department of Health (RIDOH). RIDOH produces monthly hotspot maps highlighting the top five cities/towns experiencing the highest count of nonfatal overdoses, with the data visualized on city-level maps. For the PROVIDENT trial, the RIDOH hotspot maps were accessible to all harm reduction organizations in the study. Within the trial, half of the towns were randomized to show PROVIDENT forecasting maps in addition to any data available through RIDOH hotspot maps. During focus groups, participants were able to discuss their use of the RIDOH hotspot maps as well as the PROVIDENT forecasting dashboard and the potential for their use in harm reduction outreach more broadly.

A participant described using hotspot maps retroactively to see if outreach efforts were effective:

“*So*,* as far as the map*,* I mean I think the map that they have is indicative of the work that we do. I mean they can’t have their numbers if we’re not putting them in*,* right?…So*,* if I see a map*,* and I see that the areas are shrinking*,* my heart swells. You know what I mean? That just means that we’re doing what we’re supposed to do*,*” FG 3*,* Participant 2.*

Participants in one group discussed collaborations with other agencies in order to support outreach:

“*That’s why [we] wanted to have good*,* healthy community partnerships with other agencies…because we all have the same goals and the same reasons for being there and for doing the work that we do*,*” FG 3*,* Participant 3.*

The groups reported trusting information directly from individuals in the community first and foremost, *“I mean*,* that information is the most valuable*,* is the ones on the street with the people that we’re serving*,*” FG 2*,* Participant 4.*

Some participants referenced their many years conducting peer outreach work, thus preferring their own expertise, observations, and trust in clients with whom they have strong relationships and being less inclined to use hotspot or forecasting dashboard maps to inform their outreach.

“*I mean*,* for the most part*,* we’re very knowledgeable about our community so*,* you know*,* all of us live in different parts of the area and just grazing through your own parts*,* you tend to see what’s going on…But me being here for so long*,* statistically*,* looking at a map to go out and do things the way we been doing things. The way I been in the field for so long*,* it’s just*,* I mean*,* [it’d] probably be helpful for other people*,” *FG 1*,* Participant 4.*

On an organizational level, some outreach programs are primarily directed by lived experience, including neighborhood familiarity, client testimony, and word of mouth, rather than mapping dashboards:

“*No one really follows the– it’s not that we follow the maps from Rhode Island Department of Health. It’s just good to know that when it comes out*,* that’s like a potential area to go outreach. But we’ll still do the same– we won’t change our outreach*,* what we specifically do…It doesn’t really dictate where we outreach*,* we still go to the same places*,” *FG 1*,* Participant 7.*

Participants appeared familiar with the hotspot maps as a whole and discussed the ways they apply findings from these data to actionable outreach goals. A participant expressed that hotspot mapping served the organization’s outreach efforts:

“*And then*,* there’s times where we use word of mouth*,* of course*,* so I think it’s a fair mix of using word of mouth on the street*,* listening to community members*,* getting data from [dashboards]. And sometimes*,* we’re already aware of where the hotspots are just based on our efforts*,* but seeing it represented in the data is nice because it’s confirmatory. I mean*,* it’s not great that it’s happening*,* but it’s nice to know*,* okay*,* we’ve really been working on this area because we’ve assessed that there is a need*,* so to see it reflect in the data is nice because it shows us that our efforts are where they’re supposed to be*,” *FG 2*,* Participant 4.*

Participants described monthly engagement with the PROVIDENT forecasting dashboard and the “points of interest” feature showing five locations of highest foot traffic within each census tract:

“*We’ve used [the PROVIDENT forecasting dashboard] quite a bit. I definitely used it a lot more earlier on because I spent more days in the field*,* and for me…when I was going somewhere I didn’t really know anything about*,* I would look it up to see what the top five places were and kind of*,* then go look at a map of*,* what was in that area and get an idea of where I was starting in that particular place*,” *FG 2*,* Participant 1.*

Discussion among participants highlighted the effectiveness of the forecasting dashboard in generating accurate predictions.

“*One thing that was really cool*,* it was a couple weeks ago*,* we had one of our*,* like*,* PROVIDENT things after a RIDOH meeting…there was one community that was at*,* or over*,* [the overdose] threshold that typically wasn’t*,* and in a very specific area within that region…It was something that we noticed had already been on the PROVIDENT data tool…Knowing that it was a PROVIDENT prediction was pretty cool too…I mean*,* it’s not cool*,* it sucks*,* but it showed the effectiveness of the tool*,*” FG 2.*

## Discussion

Each of the harm reduction organizations represented in the focus groups varied in their operational approaches to data collection, outreach programming, and service delivery, but core themes emerged across all three focus groups. Prior research on harm reduction organizations has similarly reported on organizational themes of limited capacity, burnout and trauma exposure, and the hidden work of building and sustaining relationships in the harm reduction environment [33–35]. These contextual factors align with our inner-context theme, noting that within-organization characteristics may limit available resources for improved data collection and intervention readiness, reduce capacity for adopting dashboard-driven resource allocation decisions, and increase the burden on some organizations and their staff. Despite this, existing outer-level factors such as community trust building, harm reduction networks, funding, and usability are factors that may contribute favorably to the implementation and uptake of the PROVIDENT forecasting dashboard. Focus group participants described how strong community partnerships laid the groundwork for their organizations to use data to expand to new geographic areas.

Applying the EPIS Exploration phase, where organizations and communities consider to what extent they imagine being ready for and needing the proposed intervention, a majority of participants expressed willingness to learn more about how to interact with data presented by a map and reported that neighborhood-level data from the PROVIDENT study team were helpful [32]. Many found forecasting dashboards to be useful, effective, and complementary to other resources, such as surveillance hotspot maps, because points of interest often overlap in the data. Participants discussed using data and the PROVIDENT forecasting dashboard to inform a more efficient outreach approach and offered the study team specific feedback on useful dashboard features. However, some participants felt the hotspot maps appealed to newer staff with less knowledge of Rhode Island neighborhoods. Other participants expressed hesitancy in applying the forecasting dashboard to their ongoing outreach efforts and used it in combination with their own judgment and experience. Several participants noted the dashboard data was not compelling enough to complement their years of expertise in the field and longstanding relationships with their clients. Taken together, such attitudes toward the intervention represent a tension between participants in deciding whether to engage fully with the intervention or not during the Preparation phase.

Our results demonstrate that operational strategies and characteristics of the intervention, such as small honoraria or improvements in technology (e.g., iPads) may not be sufficient to enable harm reduction organizations to fully engage with the PROVIDENT forecasting dashboard. The EPIS Preparation phase may identify early indicators within the implementation environment (inner and outer context factors) for improved “implementation supports,” which are key strategies to enhance readiness for the use of an intervention in the Implementation stage [32]. Examples of this may include increased data collection support and data dashboard improvements, training in trauma-informed practices and grief support for peer and outreach staff across organizations, and additional feedback for the intervention team [32]. Dedicated staffing for harm reduction organizations to engage with forecasting tools is also a consideration in resource-constrained settings. Further use of a phased approach to the implementation to support outer-context demands, such as the six-phase implementation process used by the HEALing Communities Study to increase community engagement and implementation [36], may provide key points of intervention to enhance statewide advocacy networks and create the best fit for the Sustainment phase of the implementation.

Based on our findings, we maintained monthly functionality meetings for dashboard improvements, including direct feedback from outreach workers in the field using mobile and tablet devices. Responsive viewing of the PROVIDENT dashboard on mobile devices was key to sustaining the practical use of PROVIDENT by organizations. Other significant updates to increase intervention usability included adding custom zoom options to the dashboard map (e.g. ZIP codes, distinct neighborhoods) and increasing the shared permissions of data entry forms across individual and organizational users (i.e., neighborhood assessments and six-month resource plans). We also increased data entry support for organizations by the research team, shortened Data Academy workshops, and offered remote and on-site technical assistance options for Data Academy workshops. Future directions include the development of a publicly accessible version of the PROVIDENT dashboard (i.e., without the login feature) [37]. During this process, we will further explore how to visualize real-time outreach efforts and neighborhood assessment data on the dashboard maps for efficient cross-organization information sharing (i.e. crowdsourced content). Future work will also explore the implementation of forecasting dashboards and evaluation in new regions, particularly in states or large counties that have demonstrated interest in dashboard mapping and forecasting to drive resource allocation.

## Limitations

This was a pilot qualitative implementation study within a larger parent trial based in the state of Rhode Island. Therefore, the sample size of focus groups was directly limited by the organizations participating in our study. As such, these early findings are specific to the PROVIDENT forecasting dashboard implementation process and may not be readily applicable to other regions. Furthermore, participation in the focus groups was a self-selected sample within each organization consisting largely of peer outreach workers and did not represent all perspectives or roles within the organization. Individual participant demographics and their roles within the organization were not assessed, which precluded analyses to understand whether and to what extent the different roles may have influenced dashboard engagement. Transcripts were not returned to participants for additional comment or correction of the findings. The manuscript was shared with the participating organizations prior to publication. 

## Conclusion

Novel data tools such as the PROVIDENT forecasting dashboard require careful attention to the implementation environment within harm reduction organizations and local systems of service delivery. Predictive tools offer a data-driven response that can potentially change how we use data to deliver harm reduction services and overdose prevention. The extent to which the PROVIDENT forecasting dashboard is implemented effectively within organizations will be explored in future research. Using the EPIS framework allowed us to examine early-phase multilevel factors, including the inner-contextual factors of culture and stress or trauma exposure, and the outer-context factors such as organizational networks and funding support that are needed to sustain forecasting-driven outreach work. As with any new innovation or intervention, however, attention must be paid to fully equipping our community and harm reduction partners with multi-level resources, including technology, infrastructure, funding, networking, and training, so an efficient and useful predictive dashboard can be created according to the needs, capacity, and resources of the implementation setting.

## Electronic supplementary material

Below is the link to the electronic supplementary material.

Supplementary Material 1

Supplementary Material 2

## Data Availability

No datasets were generated or analysed during the current study.

## Declarations

### Clinical trial registration

This is a smaller implementation study within a broader clinical trial, registered through US registry *clinicaltrials.gov*, trial number *NCT05096429*, registered on 2021-11-15.
