# Supplementary material for: “Sometimes I’m interested in seeing a fuller story to tell with numbers” Implementing a forecasting dashboard for harm reduction and overdose prevention: a qualitative assessment
Source: BMC Public Health. 2025 Mar 7;25:915. doi: 10.1186/s12889-025-22004-y (PMC11887322; doi:10.1186/s12889-025-22004-y)
Supplement: Supplementary file 1 — Supplementary Material 1 [file 12889_2025_22004_MOESM1_ESM.pdf]

PROVIDENT

PPHC at Brown University | people place & health collective

FOCUS GROUP GUIDE

90-minutes

Focus Group Facilitator:

1. Ensure all participants have signed the Informed Consent Form.
2. Ensure all participants are aware that the Focus Group is being recorded.
3. Start Recording.
4. Explain purpose of the PROVIDENT study, set foundation for Focus Group guidelines.
5. Cover all major topic areas using the general timeline and main prompt questions below. It is up to the facilitator to decide sub-questions and follow up questions.

[START RECORDING]

Introduction Script (~10 mins)

PROVIDENT is an exciting new research study. RIDOH and the People, Places and Health and Collective (PPHC) Team at Brown are working together to find new ways to fight overdose deaths in Rhode Island. We are testing new tools to help us predict, or forecast, future overdose patterns. We know that you are the experts, and we are grateful for your time and participation.

We want to help increase harm reduction services by using as much data and community knowledge as we can. In this focus group, we are going to ask you questions about the work you're doing in the community and how your organization

uses data and reporting. We also want to hear about what helps you do better work, and what can make this work difficult.

Our focus group will start with some broad questions, and we give you space to talk through the answers as a group. We might also move the conversation along to the next topic for timekeeping purposes. This focus group is meant to last 90 minutes from start to finish. Your participation is voluntary. You may choose not to answer certain questions if they make you feel uncomfortable.

The answers you give today will be used to better understand how you all feel about using new tools, your capacity for this work, and how we can make the Web Tool better. We will invite you to participate in a focus group every six months for the next two years, the duration of the PROVIDENT Study. Everything you share here will remain confidential and we will only report on broad themes across all of our focus groups. We will remove any identifying information from our transcripts before we analyze them, but please try not to use identifying information when possible. We ask that you all respect each other's confidentiality once you leave this space by not repeating what is shared during this session.

As a reminder, we will be recording these sessions so they can be transcribed and we may take notes during the session. We will also be asking for basic demographic data, those responses will not be linked to the recordings from these sessions.

Focus Group: Initial Session

Organizational Culture - Data (~20 mins)

Discussion prompt: Data is a huge focus for a lot of funders, and data gets a lot of attention from the Task Force and State agencies like RIDOH. The first thing we want to talk about is how your organization collects and uses data and data-tools such as maps. This will help us understand your organization's culture around data.

- **Tell us about the hotspot maps.**
    - How has your organization used or not used them?
    - What do you like or dislike about them?
    - Tell us a story when maps were useful for your work.
    - What concerns might you have about using maps?

[additional prompts below, use as needed]

-
    - How often are you getting maps from RIDOH?
    - How confident are you in your ability to interpret the maps?
    - How do you feel about using the maps to deliver services?
    - Has anyone at your organization tried creating your own maps?

- Next, let's talk about data reporting--such as reports for funders or just reporting things like naloxone kits in RedCap. (Baseline)
    - Describe the types of data your organization needs to report out and how often.
        - Does the amount of data reporting feel appropriate? (e.g., too little, too much, just right?) Can you say more about that?
        - How does data reporting impact your work providing direct services?

        - What types of computer equipment or software does your organization use? Or are you all primarily using paper reporting?
    - Tell us about how a typical monthly or quarterly report gets completed.
        - What types of oversight does your organization have for data entry and reporting?
        - When is data being entered? Same day, end of month, or end of quarter? How might that impact the quality of the reporting?
        - Does your organization have any systems or processes that have been created to make reporting easier?
        - What resources do you think might help?
- How often does your organization talk about the impact of your work in data collection and reporting? How often do you see the full results of the data you collect?
- What are things you see in your work that are not reflected in the data you collect or in the data you see from RIDOH?
- If you could change one thing about your organization when it comes to data collection and reporting, what would it be?

Testing the Web Tool (Baseline) (~15 mins)

Discussion prompt: We want to test out some forms from the PROVIDENT Web Tool. We would like to hear about your initial thoughts and reactions to these forms. This information is important to us because it let's us improve upon the tool. (show at least 1 approved sample web tool form).

The goal of the PROVIDENT Web Tool is to help us share maps with you that identify "persistent hotspots" in our state--meaning hotspots that are areas at risk for overdose for reasons more than a recent increase in overdoses. These maps will only be available for our "intervention" towns, and they will zoom in to the neighborhood level. For all

towns, you can keep using the RIDOH hotspot maps and data portal that identifies recent increases in overdoses and any other data that is typically available to you.

With the PROVIDENT Web Tool, we want to take a slightly different approach to delivering outreach and harm reduction services and we will capture neighborhood-level data in three new ways.

> The Web Tool is set up to 1.) SURVEY your chosen neighborhood or town with the quick 1pg assessment form at baseline (sample web tool form 1).
>
> Then 2.) PLAN out 1-2 ways to prioritize that neighborhood over the next 6 months with a brief goal plan form (sample web tool form 2).
>
> Finally, we want you to 3.) FOLLOW-UP once a month by logging into the Web Tool and track progress on the goal plan form (sample web tool form 2).

Our goal with PROVIDENT is to offer technical assistance training every 6 months, and RIDOH can provide additional 1-1 support on a monthly basis.

- Please offer your thoughts on this form (screen share Web Tool landing page, then go to sample web tool form 1). What information do you find helpful? What information is not helpful?
- What would make this form most useful for your work in the next 6 months?
    - RIDOH will also have access to the forms you complete, and can help you with your 6 month plans. Are there any other ways you might want RIDOH to support this work?
    - Are there any other ways you might want technical assistance or training to support this work? Examples might include another data academy series, workshops on administrative supervision, trauma informed care, burnout, etc.

Team Culture & Resources (~45 mins)

Discussion prompt: For the last topic we want to discuss the overall culture and resources for staff at your organization. As a reminder, all responses are confidential and will only be reported as larger themes across all organizations. Please respect the privacy of your fellow participants by keeping all conversations in this room.

- Talk to us about how your organization changed in the past 6 months?
  - Has funding for outreach and supplies increased or decreased?
  - How has demand for services increased or decreased?
  - Has staffing changed or largely remained the same?

- Describe the types of professional development or formal training your organization has offered for staff in the last 6 months.
  - Can you give some examples of what has been offered or suggested?
  - Are there any examples of requests from staff?
  - How do you feel about professional development?

- Talk about how your organization has or has not addressed topics such as Trauma Informed Care, Burnout, or Compassion Fatigue in the last 6 months. (facilitator, use examples if needed)
  - Examples of this at the staff-level might include clinical supervision, team based supervision, workshops in each of those topics, culture of taking intentional time off.
  - How do you feel about this as staff?

- Talk about how your organization has or has not addressed topics such as racism or structural racism in the last 6 months. (facilitator, use examples if needed)
  - Examples of this at the service level might include programs that address racialized police violence or advocacy for decriminalization.
  - Examples of this at the staff-level might include town hall discussions, acknowledgment of structural racism by leaders in an organization, or anti-racism training.
  - How do you feel about this as staff?

- How do you define harm reduction?
    - For our discussion today, when we say harm reduction we mean the way that staff believe in and show respect for people who are actively using drugs .
    - We are also referring to services that reduce the health harms of drug use such as needle exchange, fentanyl test strips, and naloxone kits.

- Describe how your organization has approached harm reduction in the last 6 months. (use examples if needed)
    - Examples of this at the service level might include the types of services you provide or where you provide them (naloxone, fentanyl test strips, or syringes; outreach to encampments, mobile delivery, etc).
    - Examples of this at the staff level might include specific support for hiring people who currently use drugs or are in recent recovery, or providing 1-1 supervision for staff.
    - Overall, how would you say that your staff feel about harm reduction?

- What do you wish your organization would prioritize in terms of training and support for staff in the next 6 months?

Facilitator: Thank you all so much for your time, honesty, and participation. We will reach out again in about 5 months to schedule our next round of focus groups and we hope you will join us again.

1. [END RECORDING]
2. DISTRIBUTE PARTICIPANT COMPENSATION
