# Supplementary material for: “Sometimes I’m interested in seeing a fuller story to tell with numbers” Implementing a forecasting dashboard for harm reduction and overdose prevention: a qualitative assessment
Source: BMC Public Health. 2025 Mar 7;25:915. doi: 10.1186/s12889-025-22004-y (PMC11887322; doi:10.1186/s12889-025-22004-y)
Supplement: Supplementary file 2 — Supplementary Material 2 [file 12889_2025_22004_MOESM2_ESM.pdf]

Identifier				Code / Sub Code Name	Definitions (Use this code to...)
**1**				**Exploration: Individual-level data skills**	**Capture characteristics of individuals within an organization related to their familiarity with and ability to interpret data**
	1.1			Knowledge of data precision	Capture the extent to which participants are equipped with the skills to assess data precision
	1.2			Knowledge of data accuracy	Capture the extent to which participants are equipped with the skills to assess data accuracy
	1.3			Understanding data-driven maps	Capture the extent to which participants know how to use and interpret data-driven maps
		1.3.1		Hotspot maps (individual level)	Capture participants' own understanding of how to use or interpret hotspot maps
		1.3.2		Other surveillance data	Capture participants' own understanding of how to use or interpret surveillance data visualizations other than maps (i.e. bar charts)
**2**				**Inner Context: Organizational capacity and culture**	**Capture characteristics of the organization's work climate**
	2.1			Organizational capacity	Capture the organization's time and staffing bandwidth
		2.1.1		Flexibility	Capture the organization's capacity to be flexible and adapt to change
	2.2			Administrative supervision	Capture the organization's approach to providing support and oversight for administrative work
	2.3			Comfort with harm reduction approach	Capture the extent to which the organization uses harm reduction interventions, lists types of interventions, or mentions increasing or decreasing harm reduction services
		2.3.1		Naloxone distribution	Describe the organization's efforts to distribute naloxone
		2.3.2		Fentanyl test strips	Describe the organization's efforts to distribute fentanyl test strips
		2.3.3		Smoking kits	Describe the organization's efforts to distribute smoking kits
		2.3.4		Needles	Describe the organization's involvement in needle exchange programs
		2.3.5		Vending machines	Describe the organization's efforts to install vending machines
	2.4			Stress/burnout among peer workers	Capture the dynamics of stress and burnout among peers who work for the organization
		2.4.1		*Burnout prevention training	Capture elements of training (i.e., when onboarding new members of organization) to provide adequate support and resources to prevent burnout
	2.5			Programmatic approach to community outreach	Capture the organization's approach to community outreach from a programmatic or administrative standpoint (less about actual relationships & engagement w/ community as in 5)
		2.5.1		Prioritizing hotspots	Capture the organization's approach to targeting community outreach to hotspots identified by either data or lived experience
		2.5.2		*Lived experience	Capture the extent to which the organization values and applies lived experience
		2.5.3		Urban vs. rural	Capture differences in the organization's approach to community outreach by urbanicity
		2.5.4		Word of mouth	Capture strategies to reach community members and share services via word of mouth (i.e., informal interpersonal conversations)
	2.6			Pandemic-related changes	Capture changes in the organization's work climate as a result of the pandemic
	2.7			Health equity	Capture the organization's approach to addressing structural racism and other systemic forms of oppression
	2.8			*Future directions	Capture future organization-level plans for outreach, data collection/reporting, etc.
**3**				**Outer Context: Organizational-level data capacity, expectations**	**Capture characteristics of the organization's data capabilities**
	3.1			Organizational culture toward data	Describe the organizational culture toward data
		3.1.1		Data entry	Describe the organization's capacity for data entry
		3.1.2		Data quality	Describe the organization's capacity for monitoring data quality
			3.1.2.1	Over/underreporting	Capture the extent to which data reporting mechanisms systematically over- or under-report data metrics
			3.1.2.2	Perceived accuracy	Capture the extent to which data align with lived experience
			3.1.2.3	Stigma	Capture the extent to which stigma serves as a barrier to accurate data reporting
			3.1.2.4	Staffing constraints	Capture the extent to which staffing constraints serve as a barrier to accurate data reporting
			3.1.2.5	Data representativeness	Capture the extent to which data are representative of the communities served by the organization
		3.1.3		Using data to drive change	Capture the extent to which the organization applies findings from data to drive change
			3.1.3.1	Data to action	Capture the extent to which the organization adopts a "data to action" approach
			3.1.3.2	*Data confirming known need	Capture the extent to which data confirms hotspots identified by community members and prior organizational efforts
			3.1.3.3	*Data confirming work well done	Capture the extent to which the organization has already deployed resources to hotspots identified by data
			3.1.3.4	Retrospective data	Captures the extent to which retrospective data hinders timely action (refers to need for data in real time)
		3.1.4		*Data usability	Captures the extent to which the organization finds data to be of use to theirs or others' outreach efforts
	3.2			Data reporting	Describe the organization's approach to data reporting
		3.2.1		Reports for funders	Describe the organization's approach to developing data reports for funders
		3.2.2		Monthly/quarterly reports	Describe the organization's approach to developing monthly/quarterly data reports
			3.2.2.1	Data reporting meetings	Describe the organization's approach to allocating time for data reporting
		3.2.3		Reporting software	Describe the organization's approach to using reporting software
		3.2.4		Limitations of data collection/reporting	Describe the organization's limitations to complete data collection and reporting
			3.2.4.1	Gaps in quantitative data	Capture the extent to which gaps in quantitative data serve as a barrier to data completeness
		3.2.5		Reporting burden	Capture the extent to which data reporting burdens organizational capacity
	3.3			Response to maps	Describe the organization's receptiveness to geographic data
		3.3.1		Hotspot maps (organizational level)	Describe the organization's approach to using hotspot maps
			3.3.1.1	Intensive mapping meetings	Capture the extent to which the organization allocates time to review hotspot maps
			3.3.1.2	Concentrating efforts in hotspots	Capture the extent to which the organization applies their findings from hotspot maps to organizational outreach efforts
	3.4			Data availability	Capture the extent to which data are made readily available to the organization
		3.4.1		EMS data	Capture references to EMS data made available via RIDOH
		3.4.2		ROAAR	Capture references to ROAAR (a public health alert from RIDOH based on non-fatal overdose data)
		3.4.3		Hospital data	Capture references to hospital data made available via RIDOH

Identifier				Code / Sub Code Name	Definitions (Use this code to...)
		3.4.4		Urban vs. rural	Capture differences in data availability by urbanicity
		3.4.5		*Social media	Capture references to social media as a data source
		3.4.6		*Proximity to data	Capture the extent to which data are made readily available to individuals depending on their role within the organization
	3.5			Data collection	Describe the organization's approach to data collection
**4**				**Characteristics of the Intervention: Web tool utilization**	**Describe how the organization uses the web tool in practice**
	4.1			Planned technical assistance	Describe efforts by the organization to build in opportunities for technical assistance and training
		4.1.1		Data academy	Capture references to PROVIDENT data academy training sessions
			4.1.1.1	Neighborhood assessment	Capture references to PROVIDENT neighborhood assessments
			4.1.1.2	Web tool planning forms	Capture references to PROVIDENT web tool planning forms
		4.1.2		Technical assistance (TA) series	Capture references to PROVIDENT TA training sessions
		4.1.3		Training/support around emotional labor, trauma exposure & outreach skills	Describe the organization's approach to training and offering support for their employees around emotional labor, trauma exposure, and outreach skills
		4.1.4		Communication	Describe the organizations' approach to communication with their employees about the web tool as well as technical assistance and training opportunities
	4.2			Web tool usability	Describe the capacity of the web tool to meet the organization's needs
		4.2.1		Helpful features	Describe helpful features of the web tool
		4.2.2		Compatibility with other resources	Describe the compatibility of the web tool with other resources
		4.2.3		Limitations of web tool	Describe the limitations of the web tool
		4.2.4		Barriers to use	Describe barriers that the organization faces to effectively using the web tool
	4.3			Web tool reliability	Describe the extent to which the organization call rely on the web tool to function effectively
**5**				**Outer-Context: Community engagement, relationships**	**Describe features of the organization's involvement and engagement with the community**
	5.1			Receptiveness to harm reduction	Capture the extent to which the community is receptive to harm reduction interventions
		5.1.1		Stigma toward drug use	Capture the extent to which the community stigmatizes and is potentially hostile toward people who use drugs
	5.2			Building rapport w/ clients	Describe the organization's approach to building trusting relationships with people who use drugs who use their services
	5.3			Building trust w/ community	Describe the organization's approach to establishing trust in the broader community
	5.4			Receptiveness to data sharing	Capture the extent to which the organization is open to sharing their data findings across organizations and with the community
	5.5			Partnerships with existing agencies	Describe the organization's existing relationships with state agencies, other organizations, etc.
	5.6			Neighborhood familiarity	Describe the organization's familiarity (from a spatial, structural, or social lens) with the neighborhoods they serve
**6**				**Administrative coding**	
	6.1			Illustrative quote	Capture poignant, representative, or compelling quotes or narratives
	6.2			Participant identity	Capture lived experience or other personal characteristics participants share that shape their identity and perspective
	6.3			Don't know	Capture text that may be important to code but requires further discussion among coders
	6.4			*Internalized stigma	Capture stigmatizing language among conversation between participants
	**Codes denoted with an asterisk and highlighted in gray emerged during the coding process*				
